# Silver Nanoparticles Modified by Gelatin with Extraordinary pH Stability and Long-Term Antibacterial Activity

**DOI:** 10.1371/journal.pone.0103675

**Published:** 2014-08-06

**Authors:** Martin Sivera, Libor Kvitek, Jana Soukupova, Ales Panacek, Robert Prucek, Renata Vecerova, Radek Zboril

**Affiliations:** 1 Department of Physical Chemistry, Faculty of Science, Palacky University, Olomouc, Czech Republic; 2 Regional Centre of Advanced Technologies and Materials, Faculty of Science, Palacky University, Olomouc, Czech Republic; 3 Department of Microbiology, Faculty of Medicine, Palacky University, Olomouc, Czech Republic; Texas A&M University Baylor College of Dentistry, United States of America

## Abstract

The potential for application of any nanoparticles, including silver nanoparticles (AgNPs), is strongly dependent on their stability against aggregation. Therefore, improvement of this parameter is a key task, especially in the case of AgNPs, because a correlation between size and biological activity has been demonstrated. In the present work, a natural stabilizer, gelatin, was investigated for the stabilization of AgNPs in an aqueous dispersion. The particles were prepared via a modified Tollens process, and the gelatin modifier was added prior to the reducing agent. The stability against aggregation of the AgNPs prepared by this method was more than one order of magnitude higher (on the basis of the critical coagulation concentration (CCC)) than that of AgNPs prepared via a similar method but without the assistance of gelatin. Their high stability against aggregation was confirmed over wide pH range (from 2 to 13) in which the particles did not exhibit rapid aggregation; such stability has not been previously reported for AgNPs. Additionally, gelatin not only fulfills the role of a unique stabilizer but also positively influences the modified Tollens process used to prepare the AgNPs. The diameter of the gelatin-modified AgNPs was substantially smaller in comparison to those prepared without gelatin. The polydispersity of the dispersion significantly narrowed. Moreover, the gelatin-stabilized AgNPs exhibited long-term stability against aggregation and maintained high antibacterial activity when stored for several months under ambient conditions.

## Introduction

The preparation and application of nanomaterials are currently hot topics in research and development. These innovative materials have strongly influenced the introduction and establishment of new processes and procedures, especially in the field of medicine, in the construction of new sensors, and in the development of environmental technologies. Silver nanoparticles (AgNPs) represent one of the most commercially interesting nanomaterials because of their high antimicrobial activity. AgNPs are used for antibacterial treatment of medical devices such as artificial heart valves, vascular catheters, and endoprostheses [1]–[3]. They have also been widely applied in the textile industry, where manufacturers modify fabrics with AgNPs and offer end-products such as antibacterially treated socks, underwear, and t-shirts [4], [5]. AgNPs are also used to modify filter cartridges in order to disinfect water [6], [7].

AgNPs are typically prepared via wet reduction methods, which are based on the reduction of a silver precursor using chemical or physical means. Silver ions can be reduced by strong reducing agents such as sodium borohydride as well as by mild reductants such as citrate or sugars [8], [9]. The reduction of silver ions can also be achieved using microwaves, ultrasound, or gamma radiation [8], [9]. Methods based on reduction using extracts from plants, algae, bacteria, or fungi, which are commonly termed “green” methods, have also been reported [9], [10].

Importantly, the application of AgNPs is, to a certain extent, limited by the common problem of dispersion instability against aggregation. This process results in the formation of large aggregates, which decreases the active surface area and, therefore, results in a significant decrease in their unique properties, such as their antimicrobial or catalytic activities. Consequently, to improve the utility of AgNPs and to facilitate their use in new applications, these particles should be stabilized. Modification/stabilization can be achieved either by electrostatic repulsion due to an electric double layer or by steric repulsion due to surface layers of polymers [11], [12]. Electrostatic stabilization is performed using charged molecules, which increase the strength of the charge barrier. For this purpose, molecules such as ionic surfactants are used; for example, the anionic surfactant sodium dodecyl sulfate (SDS) can be used in the case of particles with a negative surface charge [13], whereas cetyltrimethylammonium chloride or bromide (CTAC, CTAB) can be used in the case of particles with a positive surface charge [11], [14]. Steric stabilization can be accomplished by the adsorption of polymers onto the particle surfaces. The following synthetic polymers have been most frequently utilized: poly(vinylalcohols) (PVAs) [12], polyethylene glycols (PEGs) [15], polyacrylonitrile [16], polymethylmethacrylate [17], poly(lactic acid), [18] poly(methyl vinyl ether) [19], and poly(vinylpyrrolidones) (PVPs) [20]. Additionally, natural polymers such as chitosan [21], [22], polysaccharides, and gelatin are considered to be suitable for the stabilization of inorganic nanoparticles [12], [23]–[26]. Gelatin, which is composed of peptides and proteins and is widely used in the pharmaceutical, food, and cosmetic industries, readily interacts with metals and is highly efficient at stabilizing them because of its high molecular weight.

The object of the present study was to synthesize AgNPs in the presence of gelatin as a natural stabilizer and to evaluate its effects on the NPs. To this end, during the preparation of AgNPs, gelatin was added to the solution of silver ions complexed with ammonia, prior to the addition of a reducing agent. Because of this modification of the Tollens process, particles with much smaller diameters (5–40 nm) were synthesized and the system became more uniform than in the absence of gelatin. Because gelatin plays not only a modifying role but also a stabilizing role, the stability/instability against aggregation of the AgNPs was investigated. The gelatin-modified AgNPs exhibited extraordinary long-term stability against aggregation as well as stability over a wide pH range. Additionally, the modified/stabilized AgNPs exhibited exceptional long-term antibacterial activity, even when stored under ambient conditions.

## Materials and Methods

### Materials

Silver nitrate (99.9%, Aldrich), ammonia (25% (w/w) aqueous solution (p.a. Lach-Ner), sodium hydroxide (p.a. Lach-Ner), d-maltose monohydrate (p.a. Ridel-de-Haen), ascorbic acid (p.a. Penta), and sodium borohydride (p.a. Sigma-Aldrich) were used without purification for the preparation of AgNPs. The Tollens process was further modified by the presence of a natural polymer: gelatin (p.a. Loba Feinchemie). The aggregation study was performed with poly(diallyldimethylammonium) chloride (PDDA) (p.a. Sigma-Aldrich). All of the solutions used were prepared using demineralized water (conductivity 0.05 µS·cm^–1^) from an Aqual 29 water purification system (Merci).

### Synthetic methods

AgNPs were synthesized via a modified Tollens process [8], [27]. The initial concentrations of the reaction components were 1×10^−3^ mol·L^−1^ for AgNO_3_, 5×10^−3^ mol·L^−1^ for ammonia, and 1×10^−2^ mol·L^−1^ for sodium hydroxide, which was added to adjust the pH of the resulting reaction mixture to approximately 11.5. The concentration of the reducing agent (d-maltose, ascorbic acid, or sodium borohydride) was 1×10^−2^ mol·L^−1^. All of the reaction components were added to the reaction mixture in the mentioned order and with vigorous stirring of the reaction mixture. Gelatin solution was added prior to the addition of the reducing agent, and its concentration in the dispersion ranged from 0.0001 to 5% (w/w).

### Characterization

The average diameters and polydispersities of the NPs were determined by dynamic light scattering (DLS) using a Zeta Plus analyzer (Brookhaven) after correction for the change in viscosity due to the presence of the high-molecular-weight polymer in the final dispersion. Reference values of the AgNP diameter were obtained via analysis by TEM using the Gwidion software. The TEM images of the AgNPs were obtained on a JEM-2010 instrument (Jeol) operated at 200 kV. The UV/vis spectra, which were measured on a Specord S600 (Analytic Jena AG) spectrophotometer, were used to characterize the AgNPs in the prepared aqueous dispersions.

The stability against aggregation of the prepared AgNPs was characterized qualitatively and quantitatively. The samples studied for long-term stability were stored in plastic test tubes and were characterized by UV/vis spectrophotometry at one week intervals. Testing of the stability against aggregation based on determination of the critical coagulation concentration (CCC) was conducted by titration of the AgNP dispersions using a cationic polyelectrolyte, PDDA. Solutions of PDDA substantially destabilized the tested AgNPs at very low concentrations of PDDA. The concentration of PDDA at which rapid aggregation of the AgNPs was observed is referred to as the critical coagulation concentration (CCC) and was determined from the obtained UV/vis spectra. Experiments used to assess the pH stability of the prepared AgNP dispersions were performed via adjustment of the pH of the dispersions with HCl (acid region) or NaOH (basic region). In this manner, the pH values of the AgNP dispersions were adjusted to be in the range of 2 to 13. UV/vis spectra were measured for each sample 1 and 24 hours after the adjustment to the desired pH value. The change in the UV/vis spectra was evidence of aggregation of the AgNPs at the given pH value.

### Bactericidal assays

The antibacterial activities of the prepared AgNPs was assayed using a standard dilution micromethod, which allowed the minimum inhibitory concentrations (MICs) that stop the growth of the tested strains of bacteria and yeast to be determined. The dispersion of AgNPs was diluted into the microtiter plate in a geometric series (2-2048×) using culture medium (Mueller Hinton) and was subsequently inoculated with a standard amount of a tested microorganism. The obtained MIC values ranged from 54 to 0.05 mg·L^−1^, which can be considered bactericidal concentrations. The test organisms used were bacteria of the genera *Enterococcus*, *Staphylococcus*, *Escherichia*, *Pseudomonas*, and *Klebsiella* and yeast of the genus *Candida*. These microorganisms are standard reference strains (labeled according to Czech Collection of Microorganisms, Czech Republic).

## Results and Discussion

### Synthesis of silver nanoparticles

AgNPs were synthesized using a modified Tollens process [27], which is based on reduction of the silver ammonia complex cation Ag[NH_3_]^+^ in alkaline media using an appropriate reducing agent, e.g., reducing sugars. In our study, maltose, ascorbic acid, and sodium borohydride were used as reducing agents. We selected these reducing agents in order to use substances with different reducing powers. Maltose is a weak reducing agent and can be used in the classical Tollens process [8]. Ascorbic acid is a reducing agent with moderate reducing power that is also capable of reducing metals less electropositive than silver. For example, ascorbic acid can be used for the preparation of copper nanoparticles [28]. Sodium borohydride is one of the strongest reducing agents currently available for common applications. Its reducing power is so great that it can reduce iron salts to iron nanoparticles [29]. The entire process of AgNP synthesis was modified by the addition of gelatin, which is a biocompatible and biodegradable natural polymer. In this study, gelatin was used not only as a stabilizer for the prepared AgNPs but also to modify the NP growth via its strong adsorption onto the surfaces of the emerging nanoparticles.

In the case of the system where maltose was used as the reducing agent, stable AgNPs were obtained only at low concentrations of gelatin, i.e., in the range 0.0001–0.1% (w/w). Because maltose is a weak reducing agent, the reaction proceeded slowly at laboratory temperature. The reaction time was approximately 20 minutes at the lowest tested concentration of gelatin (0.0001% (w/w)), and it was prolonged to 24 hours for the highest tested concentration of gelatin (0.1% (w/w)). For this reason, experiments with gelatin concentrations greater than 0.1% (w/w) were not performed using maltose reduction. For the case of ascorbic acid as the reducing agent, stable NPs were prepared in the presence of higher concentrations of gelatin, ranging from 0.01 to 2.5% (w/w). This reducing agent proved to be sufficiently strong for reduction of the silver ammonia complex cation in the presence of the highest concentration of gelatin in this study, i.e., 2.5% (w/w). At this gelatin concentration, the reaction time was approximately 10 minutes. However, in contrast to maltose, stable AgNPs were not obtained at concentrations less than 0.01% (w/w) of gelatin because the NPs quickly aggregated into larger objects. The third reducing agent tested was sodium borohydride. This reagent possesses rather strong reducing power. As a result, the reduction was completed within 1–2 minutes. Similar to the system with ascorbic acid, when sodium borohydride was used as the reducing agent, the AgNPs were not stable at gelatin concentrations less than 0.01% (w/w).

**Figure 1:**
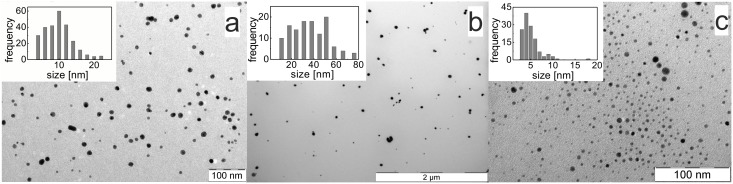
TEM images and corresponding particle size distribution histograms of AgNPs reduced by maltose (a), ascorbic acid (b), and sodium borohydride (c) in the presence of gelatin. The concentration of gelatin was 0.025% (w/w).

The prepared AgNPs were characterized primarily by dynamic light scattering (DLS) and UV/vis spectroscopy. The data concerning AgNP diameter obtained from the DLS measurements were significantly dependent not only on the reducing agent used but also on the amount of gelatin present in the system. The observed trends were the same for all three reducing agents tested: the diameter of the AgNPs increased with increasing gelatin concentration in the NP dispersion. In the cases of the maltose, ascorbic acid, and sodium borohydride reduction systems, the values of the NP diameters obtained from DLS measurements ranged from 40 to 300 nm, 70 to 400 nm, and 50 to 350 nm, respectively. However, for all of the prepared AgNP dispersions, the UV/vis spectra revealed a strong absorption plasmon peak at 410 nm, which is characteristic of AgNPs in the diameter of only several tens of nanometers. The presence of such small AgNPs in the aqueous dispersions was confirmed by transmission electron microscopy (TEM). The selected TEM images of the NPs prepared using the different reducing agents are presented in Figure 1 (and Figures S1, S2, and S3). Therefore, the true sizes of the prepared AgNPs were determined from these TEM images (Table 1).

**Table 1:** Average values of AgNP diameter determined from DLS measurements and from TEM images.

	Gelatin concentration [% w/w]	Average particle diameter [nm]* ^1^	Average particle diameter [nm]* ^2^
Maltose	0.00025	43	30.4
	0.00250	50	22.8
	0.02500	62	9.3
Ascorbic acid	0.025	70	38.3
	0.250	76	32.7
	2.500	280	17.9
Sodium borohydride	0.025	93	5.0
	0.250	133	6.5
	2.500	300	5.3

*^1^determined from DLS;

*^2^determined from TEM.

AgNPs were prepared via a modified Tollens process using various reducing agents in the presence of gelatin.

The values presented in Table 1 not only indicate that the DLS method is inappropriate for the determination of the diameter of the synthesized AgNPs in reaction systems with gelatin but also show that DLS cannot be used to determine trends related to the change in the polymer concentration. According to the TEM images, the diameter of the AgNPs prepared using maltose as the reducing agent decreased with increasing gelatin concentration from 30 nm (0.00025% (w/w) gelatin) to 9 nm (0.025% (w/w) gelatin). The same trend in diameter decrease was also observed when ascorbic acid was used as the reducing agent. Only in the system where sodium borohydride was used as the reducing agent the diameter of the prepared AgNPs did not significantly change with the change in gelatin concentration. However, in this case, the smallest NPs were prepared, with a diameter of approximately 5 nm.

**Figure 2:**
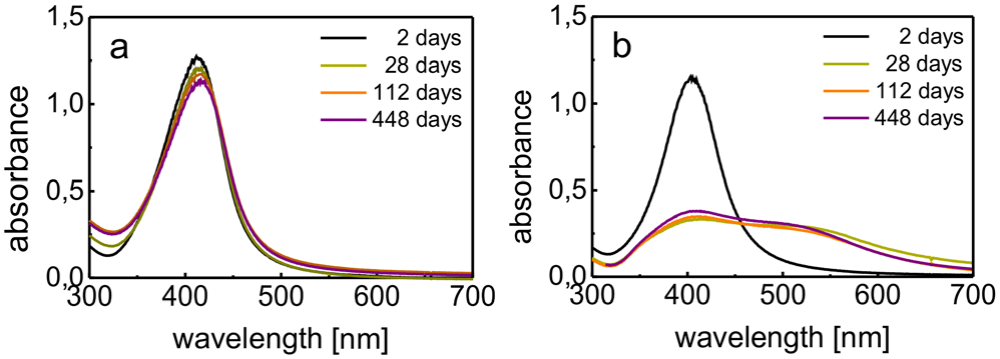
UV/vis absorption spectra of AgNPs reduced by maltose (a) and sodium borohydride (b) and modified by gelatin at a concentration of 0.01% (w/w) in the reaction system.

Long-term stability against aggregation, aside from other parameters such as the NP diameter and morphology, represents a key requirement for the possible application of any nanomaterial. An outstanding stabilization effect of gelatin on AgNP dispersions was demonstrated by the stability study based on UV/vis spectroscopy. The absorption spectra of AgNP dispersions are very sensitive to changes in the diameter of the NPs, e.g., changes caused by aggregation of the AgNPs. Therefore, all stability studies performed in this work were evaluated using UV/vis spectroscopy. To study the long-term stability against aggregation of the prepared AgNPs, the samples were stored in plastic tubes under ambient temperature in the absence of light and UV/vis spectra were recorded regularly in one week intervals for eighteen months. In the systems where maltose and ascorbic acid were used as the reducing agents, the long-term stability against aggregation was not affected by different concentrations of gelatin in the NP dispersions. No significant changes in the measured UV/vis spectra were detected over the time period tested (Figure 2a). Thus, the dispersions can be considered to be stable against aggregation. The AgNPs prepared using sodium borohydride as the reducing agent were aggregation resistant when the gelatin concentration ranged from 0.025 to 2.5% (w/w). However, the lowest tested gelatin concentration (0.01% (w/w)) was not sufficient to stabilize the AgNPs. The abrupt change in the absorption spectra after the first 7 days of storage (the absorption at 410 nm decreased, and a secondary maximum was formed at a wavelength of 540 nm) indicated that the NPs aggregated (Figure 2b). However, this semi-aggregated system remained stable for a prolonged period.

To evaluate the stability against aggregation of the AgNPs prepared here, in which gelatin provides electrostatic stabilization, a previously reported method based on titration of the prepared AgNP dispersions with a solution of the cationic polyelectrolyte PDDA was used [11]. This polyelectrolyte is suitable for investigating the aggregation of negatively charged nanoparticles and considerably destabilizes dispersions of AgNPs at very low concentrations. The changes in the AgNP dispersions, caused by the aggregation of the NPs, were observed again using UV/vis spectroscopy. From these experiments, the values of critical coagulation concentration (CCC) were determined (Figure S4). Because of the different concentration range of gelatin in the system, only one gelatin concentration (0.025% (w/w)) for all three reducing agents was chosen and the reported values of CCC were obtained as the average from four titrations. For AgNPs prepared via reduction by maltose, ascorbic acid, and sodium borohydride, the critical coagulation concentrations of PDDA were 3.9×10^−4^% (w/w), 4.5×10^−4^% (w/w), and 4.2×10^−4^% (w/w), respectively. The value of CCC for an unmodified dispersion of AgNPs was also determined and was 2.5×10^−5^% (w/w). A comparison of these results leads to the conclusion that the stabilizing effect of gelatin on the AgNPs even at a low concentration, led to an approximately fifteen-fold increase in the CCC compared to that of non-stabilized dispersions.

**Figure 3:**
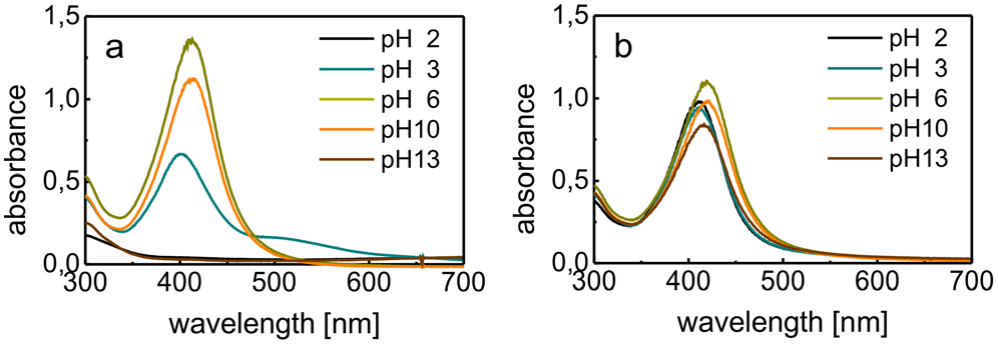
UV/vis absorption spectra of the unmodified (a) and gelatin-modified (b) AgNPs reduced by maltose at various pH values.

The potential for applications of AgNPs with respect to the maintenance of their beneficial antibacterial activity requires stability against aggregation over a wide pH range. Biological availability in *in* *vivo* studies of the bioactivity and toxicity of AgNPs is influenced by pH [2], [30]. Additionally, the toxicity of AgNPs, not only due to digestion by a living organism but also in the environment, is fundamentally influenced by the pH of the environment [31], [32]. Unfortunately, complex studies on the pH stability of aqueous dispersions of AgNPs have not been frequently reported in the literature, although poor stability of AgNPs dispersions in acidic media has been reported, especially at pH values less than 5 [33], [34]. Therefore, the stability against aggregation of gelatin-modified AgNPs prepared in this study was studied over the pH range of 2 to 13. The stability against aggregation was evaluated on the basis of changes in the surface plasmon absorption peak, which were induced by the initiation of AgNP aggregation, as in the previous stability studies. This study was performed with AgNPs prepared using maltose as the reducing agent and gelatin as the modifying/stabilizing agent at a concentration of 0.05% (w/w). An unmodified AgNP dispersion prepared using the same reducing agent was tested for comparison. The pH was adjusted by the addition of either sodium hydroxide solution (for alkaline pH conditions) or hydrochloric acid (for acidic pH conditions). The absorption spectra of all of the prepared samples were measured two hours after the pH was adjusted and again after 24 hours and one week of storage at the adjusted pH. The fundamental aggregation of AgNPs was evaluated on the basis of the disappearance of the surface plasmon absorption peak from the spectra (Figure 3). These measurements indicated a unique stability against aggregation of the gelatin-stabilized AgNP dispersion over a wide pH range, which was in contrast to the rather poor results obtained for the non-modified dispersion. In first two hours, the stability against aggregation of the modified AgNPs dispersion was observed over a remarkably wide pH range (from 2 to 13). After 24 hours, aggregation proceeded, particularly in acidic media, and the range of stability against aggregation was narrowed to a range from pH 3 to 13. After one week of storage, the range was decreased to pH values from 4 to 13. However, in the tests on unmodified AgNP dispersions, the aggregation-stable regions were rather narrow from the beginning: 3–12 for first two hours, 4–12 for first 24 hours, and 6–12 for one week of storage. Interestingly, the stability against aggregation was lower at acidic pH than at basic pH. This observation, which corresponds to the results of previous studies, is most likely related to the electrochemical behavior of gelatin. Gelatin is an amphiphilic molecule with an isoelectric point at a pH value of approximately 5 [35]. The zeta potential of AgNPs in an aqueous dispersion without gelatin was −36 mV (pH 11.5). Measurement of the zeta potential of gelatin-modified AgNPs, however, is rather complicated because of the same obstacles described for determination of the particle diameter by DLS. In alkaline media, the AgNPs should exhibit, minimally, the same zeta potential as unmodified AgNPs. However, if the pH is decreased by the addition of hydrochloric acid, the dissociation of carboxylic functional groups also decreases and the protonation of amino functional groups increases. At pH 5 (i.e., the isoelectric point of gelatin), the ionization of both types of groups is approximately equal, and protonation dominates at pH values less than 5. Therefore, the positive charge of the protonated amino groups neutralizes the negative surface charge of the AgNPs, which are less stable against aggregation compared to when the pH is greater than the isoelectric point of gelatin. Nevertheless, gelatin is a much better stabilizer than citrate; some synthetic polymers, e.g., PVP; and some sulfur-containing molecules, such as cysteine [33], [34].

### Bactericidal assays

**Figure 4:**
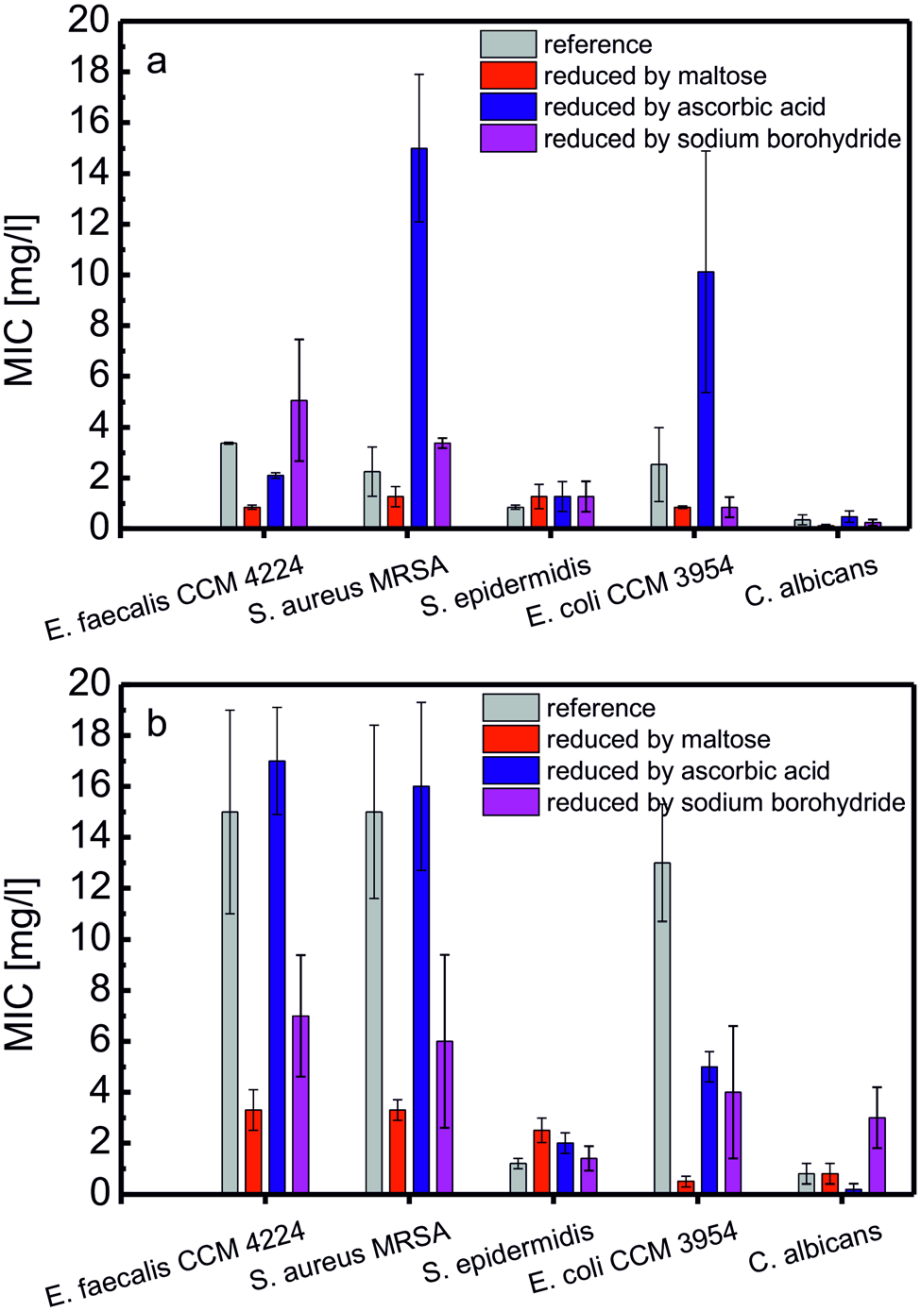
Minimum inhibitory concentrations of the AgNPs reduced by maltose, ascorbic acid, and sodium borohydride in systems influenced by the presence of gelatin at a concentration of 0.05% (w/w). Determined after one week (a) and after three months (b).

The standard dilution micromethod was applied to test the antimicrobial activity of the prepared AgNPs. The MICs of the AgNPs against both gram-negative and gram-positive bacteria and also against selected yeasts are summarized as average values from 3 independent measurements; the results are presented in Figure 4 (selected values) and in Table S1. The initial concentration of AgNPs was 108 mg·L^−1^ after preparation, and the tested dilutions ranged from twofold to 2048-fold. The AgNPs were tested one week and 3 months after preparation.

For AgNPs prepared via reduction by maltose or ascorbic acid with increasing concentrations of gelatin in the dispersions, the MIC values decreased, indicating antibacterial activity of the NPs at higher gelatin concentrations. These findings, along with the fact that the MIC values for AgNPs prepared via maltose reduction were significantly better, confirm the previously reported high antimicrobial activity of smaller AgNPs. Size effects also likely explain why the MIC values obtained for AgNPs prepared via sodium borohydride reduction were approximately independent of the gelatin concentration for the majority of the tested microorganisms. However, for some bacterial strains, the MIC values were significantly greater than for larger AgNPs prepared via maltose reduction. After 3 months of storage, the antimicrobial activity of AgNPs decreased by a factor of approximately 2–4; in addition, the decrease of antimicrobial activity was substantially greater for unmodified AgNPs compared to AgNPs prepared in the presence of gelatin.

## Conclusions

Both the fundamental particle characteristics and the stability against aggregation of AgNPs were improved through the use of gelatin as a modifier/stabilizer. This statement is no doubt valid for all of the investigated systems, i.e., for systems where maltose, ascorbic acid, or sodium borohydride were used as the reducing agent. In the presence of gelatin, a decrease in the particle diameter was observed for the system reduced by maltose (from 30 to 10 nm) and ascorbic acid (from 40 to 18 nm). In the case of AgNPs prepared via borohydride reduction, the particles were very small (approximately 6 nm) and their diameter was independent of the gelatin concentration. The gelatin-modified dispersions of AgNPs were observed to exhibit long-term stability against aggregation (minimally 18 months) and also aggregation resistance over a wide pH range (from 2 to 13). As a result, the gelatin-modified AgNPs maintained high antimicrobial activity against many bacteria and yeast species for a period of 3 months after their preparation, without requiring any special storage conditions.

## Supporting Information

Figure S1 TEM images and corresponding particle size distribution histograms of AgNPs reduced by maltose in the presence of gelatin. The concentrations of gelatin were 0.00025 (a), 0.0025 (b), and 0.025% (w/w) (c).
(DOC)

Figure S2 TEM images and corresponding particle size distribution histograms of AgNPs reduced by ascorbic acid in the presence of gelatin. The concentrations of gelatin were 0.025 (a), 0.25 (b), and 2.5% (w/w) (c).
(DOC)

Figure S3 TEM images and corresponding particle size distribution histograms of AgNPs reduced by sodium borohydride in the presence of gelatin. The concentrations of gelatin were 0.025 (a), 0.25 (b), and 2.5% (w/w) (c).
(DOC)

Figure S4 Evaluation of the CCC value from UV/vis spectra using titration with a PDDA solution.
(DOC)

Table S1 Minimum inhibitory concentrations [mg·L^−1^] of AgNPs reduced by maltose, ascorbic acid, or sodium borohydride in systems influenced/modified by gelatin at a concentration of 0.05% (w/w).
(DOC)
