# Supplementary material for: Silver Nanoparticles Modified by Gelatin with Extraordinary pH Stability and Long-Term Antibacterial Activity
Source: PLoS One. 2014 Aug 6;9(8):e103675. doi: 10.1371/journal.pone.0103675 (PMC4123891; doi:10.1371/journal.pone.0103675)

**Figure S1** TEM images and corresponding particle size distribution histograms of the AgNPs reduced by maltose in the presence of gelatin. The concentrations of gelatin were 0.00025 (a), 0.0025 (b) and 0.025 % w/w (c).


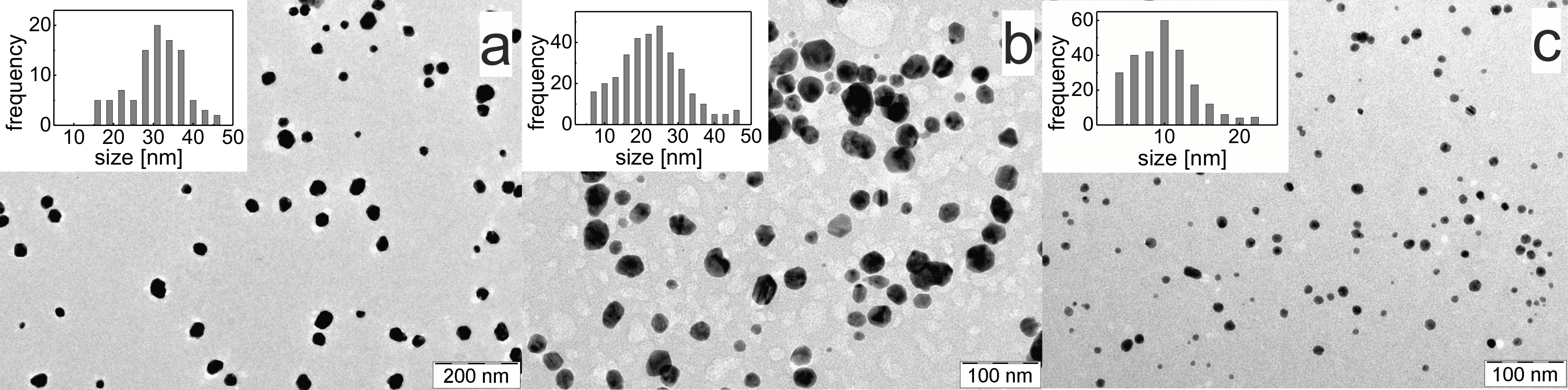

Supplement: Figure S1 — TEM images and corresponding particle size distribution histograms of AgNPs reduced by maltose in the presence of gelatin. The concentrations of gelatin were 0.00025 (a), 0.0025 (b), and 0.025% (w/w) (c). (DOC) [file pone.0103675.s001.doc]
