# Supplementary material for: Silver Nanoparticles Modified by Gelatin with Extraordinary pH Stability and Long-Term Antibacterial Activity
Source: PLoS One. 2014 Aug 6;9(8):e103675. doi: 10.1371/journal.pone.0103675 (PMC4123891; doi:10.1371/journal.pone.0103675)

**Figure S2** TEM images and corresponding particle size distribution histograms of the Ag NPs reduced by ascorbic acid in the presence of gelatin. The concentrations of gelatin were 0.025 (a), 0.25 (b) and 2.5 % w/w (c).


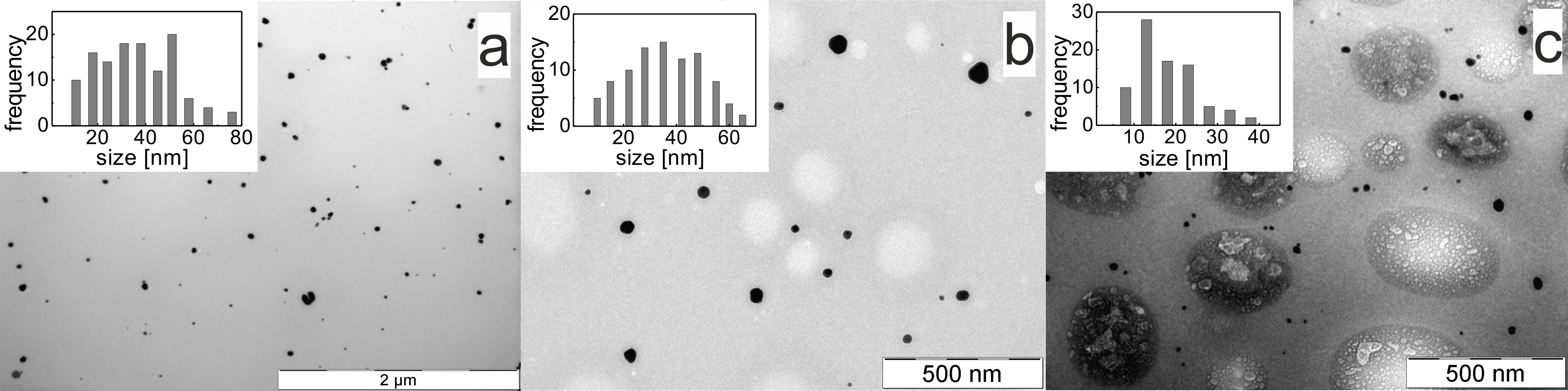

Supplement: Figure S2 — TEM images and corresponding particle size distribution histograms of AgNPs reduced by ascorbic acid in the presence of gelatin. The concentrations of gelatin were 0.025 (a), 0.25 (b), and 2.5% (w/w) (c). (DOC) [file pone.0103675.s002.doc]
