# Supplementary material for: Silver Nanoparticles Modified by Gelatin with Extraordinary pH Stability and Long-Term Antibacterial Activity
Source: PLoS One. 2014 Aug 6;9(8):e103675. doi: 10.1371/journal.pone.0103675 (PMC4123891; doi:10.1371/journal.pone.0103675)

**Figure S3** TEM images and corresponding particle size distribution histograms for the AgNPs reduced by sodium borohydride in the presence of gelatin. The concentrations of gelatin were 0.025 (a), 0.25 (b) and 2.5 % w/w (c).


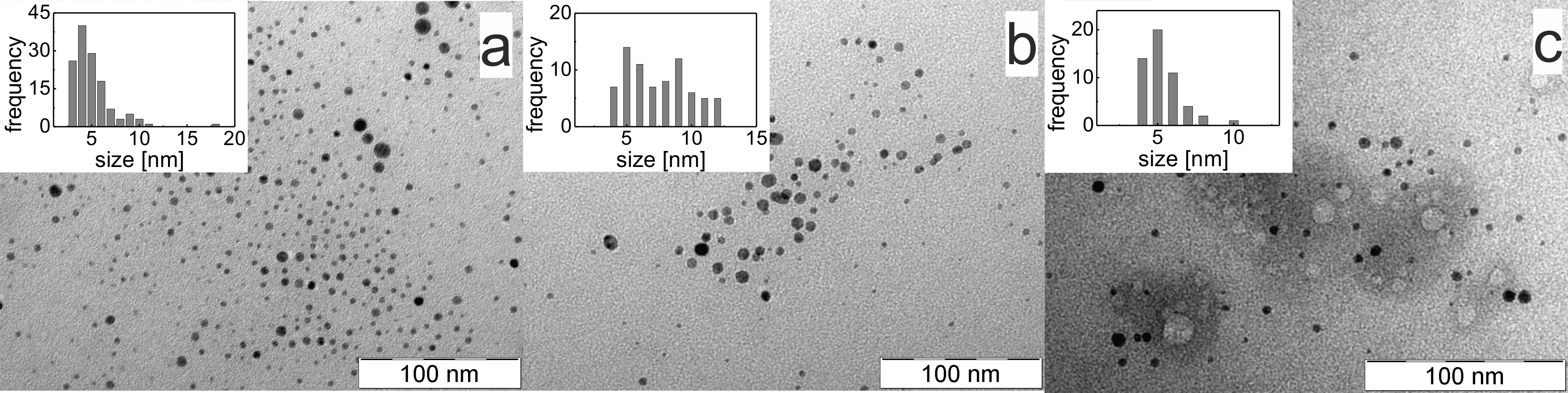

Supplement: Figure S3 — TEM images and corresponding particle size distribution histograms of AgNPs reduced by sodium borohydride in the presence of gelatin. The concentrations of gelatin were 0.025 (a), 0.25 (b), and 2.5% (w/w) (c). (DOC) [file pone.0103675.s003.doc]
