# Supplementary material for: Silver Nanoparticles Modified by Gelatin with Extraordinary pH Stability and Long-Term Antibacterial Activity
Source: PLoS One. 2014 Aug 6;9(8):e103675. doi: 10.1371/journal.pone.0103675 (PMC4123891; doi:10.1371/journal.pone.0103675)

**Figure S4** Illustrative evaluation of CCC value from UV/vis spectra using titration method with PDDA solution.


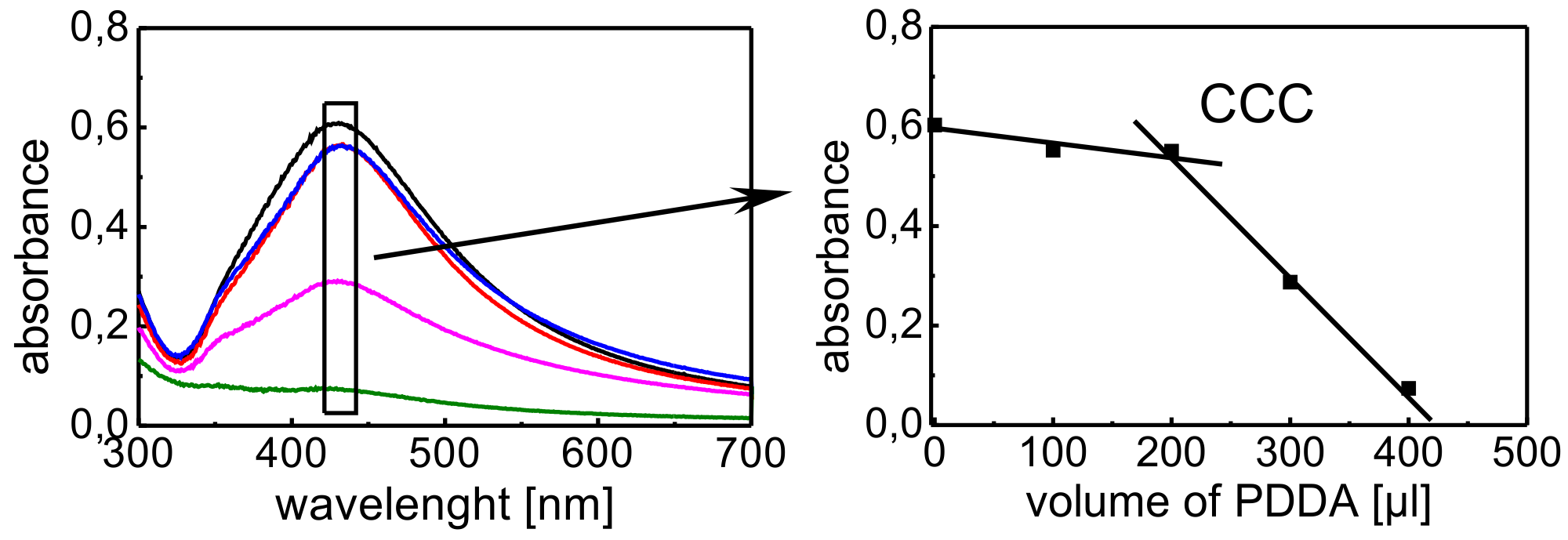

Supplement: Figure S4 — Evaluation of the CCC value from UV/vis spectra using titration with a PDDA solution. (DOC) [file pone.0103675.s004.doc]
