# Supplementary material for: Silver Nanoparticles Modified by Gelatin with Extraordinary pH Stability and Long-Term Antibacterial Activity
Source: PLoS One. 2014 Aug 6;9(8):e103675. doi: 10.1371/journal.pone.0103675 (PMC4123891; doi:10.1371/journal.pone.0103675)
Supplement: Table S1 — Minimum inhibitory concentrations [mg·L−1] of AgNPs reduced by maltose, ascorbic acid, or sodium borohydride in systems influenced/modified by gelatin at a concentration of 0.05% (w/w). (DOC) [file pone.0103675.s005.doc]

**Table S1** Minimum inhibitory concentrations of the Ag NPs reduced by maltose, ascorbic acid or sodium borohydride in systems influenced/modified by gelatin in concentration 0.05 % w/w.

|  | **Maltose** | | **Ascorbic acid** | **Sodium borohydride** |
| --- | --- | --- | --- | --- |
| **Microorganism** | Ref. (without gelatin) | 0.05 % gelatin | 0.05 % gelatin | 0.05 % gelatin |
| Enterococcus faecalis CCM 4224 | 3.37 ± 0,04 | 0,84 ± 0,08 | 2,25 ± 0,11 | 5.06 ± 2,39 |
| Staphylococcus aureus CCM 3953 | 2.81 ± 0,97 | 1.26 ± 0,49 | X | 6.75 ± 0,55 |
| Escherichia coli CCM 3954 | 2.53 ± 1,46 | 0.84 ± 0,05 | 10.12 ± 4,77 | 0.84 ± 0,42 |
| Pseudomonas aeruginosa CCM 3955 | 3.37 ± 0,15 | 0.84 ± 0,05 | X | 1.26 ± 0,60 |
| Pseudomonas aeruginosa | 2.25 ± 0,94 | 2.10 ± 1,29 | 6.75 ± 0,20 | 1.68 ± 0,68 |
| Staphylococcus epidemidis 1 | 1.40 ± 0,49 | 1.26 ± 0,49 | 1.26 ± 0,59 | 2.53 ± 0,88 |
| Staphylococcus epidemidis 2 | 0.84 ± 0,08 | 1.26 ± 0,49 | 1.26 ± 0,59 | 1.26 ± 0,60 |
| Staphylococcus aureus MRSA | 2.25 ± 0,97 | 1.26 ± 0,40 | 15.12 ± 2,90 | 3.37 ± 0,2 |
| Enterococcus faecium VRE | 2.81 ± 0,91 | 1.26 ± 0,49 | 30.37 ± 4,52 | 5.06 ± 2,30 |
| Klebsiella pneumoniae ESBL | 3.37 ± 0,05 | 0.84 ± 0,04 | X | 2.53 ± 1,19 |
| Candida albicans 1 | 0.35 ± 0,20 | 0.10 ± 0,06 | 0.47 ± 0,22 | 0.23 ± 0,12 |
| Candida albicans 2 | 0.35 ± 0,16 | 0.26 ± 0,18 | 0.47 ± 0,20 | 0.84 ± 0,33 |
| Candida tropicalis | 0.28 ± 0,12 | 0.47 ± 0,11 | 0.84 ± 0,28 | 1.26 ± 0,55 |
| Candida parapsilosis | 0.45 ± 0,21 | 0.15 ± 0,06 | 0.63 ± 0,29 | 0.31 ± 0,15 |
